# Individual and combined contamination of oxytetracycline and cadmium inhibited nitrification by inhibiting ammonia oxidizers

**DOI:** 10.3389/fmicb.2022.1062703

**Published:** 2022-12-01

**Authors:** Xiaoxu Cao, Wei Zhao, He Zhang, Jitong Lin, Jingying Hu, Yanhong Lou, Hui Wang, Quangang Yang, Hong Pan, Yuping Zhuge

**Affiliations:** National Engineering Research Center for Efficient Utilization of Soil and Fertilizer Resources, College of Resources and Environment, Shandong Agricultural University, Tai’an, China

**Keywords:** oxytetracycline, cadmium, nitrification, ammonia-oxidizing archaea, ammonia-oxidizing bacteria

## Abstract

**Introduction:**

The large-scale development of animal husbandry and industrialization lead to more and more serious co-contamination from heavy metals and antibiotics in soils. Ecotoxic effects of residues from antibiotics and heavy metals are of increasing concern.

**Materials and Methods:**

In this study, oxytetracycline (OTC) and cadmium (Cd) were selected as target pollutants to evaluate the individual and combined effects on nitrification process using four different soil types sampled from North to South China through a 56-day incubation experiment.

**Results and Discussion:**

The results demonstrated that the contaminations of OTC and Cd, especially combined pollution had significant inhibitory effects on net nitrification rates (NNRs) as well as on AOA and AOB abundance. The toxic effects of contaminants were greatly enhanced with increasing OTC concentration. AOB was more sensitive than AOA to exogenous contaminants. And the interaction effects of OTC and Cd on ammonia oxidizers were mainly antagonistic. Furthermore, Cd contaminant (with or without OTC) had indirect effects on nitrification activity via inhibiting mineral N and AOA/AOB, while OTC alone indirectly inhibited nitrification activity by inhibiting ammonia oxidizers. The results could provide theoretical foundation for exploring the eco-environmental risks of antibiotics and heavy metals, as well as their toxic effects on nitrification processes.

## Introduction

Antibiotics have become important antimicrobial drugs in recent years, and have been widely applied in medicine and agriculture for disease treatment and health protection. However, antibiotics are incompletely metabolized by humans or animals, and most antibiotics are excreted into the environment, leading to increase in antibiotic resistant bacteria (ARB) and antibiotic-resistant genes (ARGs; Knapp et al., 2010; Li et al., 2012; Wang et al., 2019b). China was a major producer and consumer of antibiotics and approximately 53,800 tons of different antibiotics were imported into environment only in 2013 (Zhang Q.-Q. et al., 2015). The worldwide consumption of antimicrobials was expected to increase from 631,511,560 to 105,596 3,605 tons from 2010 to 2030, and the consumption of antibiotics in Brazil, Russia, India, China, and South Africa would increase by 99% (Van Boeckel et al., 2015). Veterinary antibiotics were commonly used to improve animal growth efficiency. The tetracyclines such as tetracycline, oxytetracycline and chlortetracycline were widely applied to animal feed in many countries. All these contaminants tended to be excreted into the environment as part of animal excrement, such as manure and urine, and agricultural organic fertilizers (Sarmah et al., 2006; Zhang H. et al., 2015; Cao et al., 2016; Li et al., 2019; Guo et al., 2021).

Oxytetracycline (OTC) is one of the most widely used antibiotics in the world and is widely applied as feed additive in animal husbandry to stimulate animal growth (Qin et al., 2019). Besides, OTC has various functional groups, which are easily combined with soil cations or metal oxides, and thus could be adsorbed on the soil surface by cation exchange, hydrogen bond exchange, surface ligand chelation. Researchers found that the adsorption curve of OTC fit the Freundlich model, with adsorption rates close to 100%, indicating that the soil had high affinity for OTC (Tolls, 2001; Mifflin et al., 2006; Pan and Chu, 2016; He et al., 2019; Conde-Cid et al., 2020; Guo et al., 2021). In addition, some studies found that only small portions of tetracycline were ingested by animals, and nearly 60%–90% of tetracycline was released into the environment, including mud, soil and water (Liu et al., 2020). Antibiotics have direct or indirect effects on soil microbial communities, damaging the structure and function of microbial communities in various ways, resulting in the inhibition and even extinction of certain microorganisms involved in critical ecological functions (Grenni et al., 2018).

Heavy metal (loid)s contaminants in soils and plants harm food safety and threaten human health (Qin et al., 2021). Furthermore, heavy metals cannot be biodegraded in the environment, which will persist in nature and cause serious environmental challenges (Leong and Chang, 2020). Rapid industrialization and inadequate waste management have caused high incidences of toxic metal pollution in Africa and other developing countries (Okereafor et al., 2020). Similarly, the lack of effective environmental management in some nonferrous metal smelters resulted in the emissions of heavy metals in the surrounding environment and caused crop heavy metal concentrations far above national maximum allowance concentrations (Cai et al., 2019; Wang et al., 2019a).

Cadmium (Cd) was representative toxic metal that affected soil nutrient cycle and plant growth (Clemens and Ma, 2016; Rahman and Singh, 2019). Moreover, based on the results of widespread database evaluation of heavy metal concentrations in industrial and agricultural land soils, Cd, Pb and As were selected as priority control heavy metals (Yang et al., 2018). Cd was mainly existed as CdCO_3_ (high pH) and CdS (low pH) in soils these complexes could be transformed and be absorbed by plants, and eventually threatened human health by accumulating through the food chain (He et al., 2015; Haider et al., 2021). Huang et al. (2019) found continuous increase of soil Cd and Hg concentration in industrial areas countrywide by analyzing data published from 2005 to 2017. Hu et al. (2020) proposed that heavy metal levels exceeded national environmental background limits and increased over time in most Chinese provinces. Previous studies found that Cd was mainly derived from human activities such as mining, metallurgy, wastewater irrigation, and fertilization. Fertilizer, in particular, was found to be the major source of Cd (Shi et al., 2019). Meanwhile, the levels of heavy metals and antibiotics in organic fertilizers were significantly higher than environmental background concentrations (Zhu et al., 2013). It has been reported that metal contamination as long-term and widespread persistent selection stress could transmit antibiotic resistance genes (ARG; Baker-Austin et al., 2006; Guo et al., 2021).

Nitrogen (N) is crucial component and nutrient for the growth and development of plants. N availability depends on various chemical processes catalyzed by microorganisms, thus microorganisms play important role in N cycle (Kuypers et al., 2018). Nitrification is a microbially regulated conversion process of ammonia to nitrate *via* nitrite, which is essential for the global N cycle. Of which, ammonia oxidation is the first step and also rate limiting step of nitrification performed by ammonia-oxidizing bacteria (AOB) and ammonia-oxidizing archaea (AOA; Galloway et al., 2008; Beeckman et al., 2018; Pan et al., 2018). Recently, microorganisms capable of complete oxidation of ammonia to nitrate (comammox) have been discovered and radically challenged the conventional concept of two-step nitrification (Daims et al., 2015). The ammonia monooxygenase subunit A (*amoA*) gene can be used as molecular marker, which was detected in many environments and widely applied to analyze the species, abundance, community structure and evolutionary relationships of ammonia-oxidizing microorganisms (Wang J. et al., 2018; Aigle et al., 2019; Pan et al., 2021).

Previous studies have extensively reported the adverse effects of antibiotics and heavy metals on soil microorganisms and ecosystem function (Wang J. et al., 2018; Wang L. et al., 2018; Tang et al., 2020). For example, OTC inhibited protein synthesis by disrupting amino acid chain elongation at the 30S subunit of the ribosome, leading to decreased nitrification rate and bacterial growth (Kong et al., 2006). The soil microbial community function showed pronounced negative effect with increasing concentrations of OTC (Kong et al., 2006; Ma et al., 2016). Likewise, Cd exposure significantly decreased ammonification, potential nitrification activity, denitrification, as well as the *amoA* gene abundance and soil urease activity, which exerted a negative impact on N cycle processes (Chen et al., 2001; Bai et al., 2021; Lu et al., 2022). Zhao et al. (2020) also found that Cd inhibited soil nitrification by eliminating AOB in acidic ferrosol. Tang et al. (2020) demonstrated that addition of heavy metals alone or combined with OTC inhibited NH_4_^+^ oxidation to NO_2_^−^, then reduced the concentration of NO_3_^−^-N in sandy loam and clay loamy soils.

Antibiotics and heavy metals commonly coexisted in the soil, thus making contamination more widespread and complex (Guo et al., 2018). OTC contained several O-functional groups, which could act as potential electron donors and complexation with metal ions (Guo et al., 2021; Zhu et al., 2022). The interactions between antibiotics and heavy metals affected each other’s environmental behavior and toxicological effects (Zhao et al., 2013). In addition, OTC and Cd showed dual effects on nitrification by promoting or inhibiting the growth and activity of ammonia oxidizing microorganisms. Bai et al. (2021) found that Cd addition at 15 mg·kg^−1^ promoted N mineralization rates and net N mineralization accumulation, while Cd addition at 100 mg·kg^−1^ inhibited N transformation in constructed wetland soils. Chen et al. (2001) discovered that Cd addition at 2 and 5 mg·kg^−1^ could stimulate nitrification while Cd addition at 10 and 20 mg·kg^−1^ inhibited nitrification in alluvial soils. Moreover, Wang L. et al. (2018) found that AOA abundance was promoted at 23 mg·kg^−1^ of OTC addition and was inhibited at 92 and 368 mg·kg^−1^ of OTC addition in brown soils. Besides, Zhao et al. (2021) reported that Cd (4 and 8 mg·kg^−1^) directly stimulated AOB-dominated nitrification at high NH_4_^+^ levels, leading to acceleration of N transformation in clay soils. He et al. (2018) found that AOB was more sensitive than their AOA counterparts to Cu pollution in fluvo-aquic soils. Similarly, soil nitrification activity was inhibited under the threat of lead (Pb) and Cd contamination in silty clay soils. However, Wang et al. (2019) showed that single and combined addition of sulforaphane (SM2) and Cu had persistent and significant synergistic inhibitory effects on AOB and antagonistic inhibitory effects on AOA in brunisolic soils. The toxic effects of antibiotics and heavy metals on nitrification depended on a series of factors including soil organic matter (SOM), pH, clay minerals, inorganic anions, cations, as well as the contaminants concentration (Tang et al., 2020).

Therefore, four representative soils from north to south of China varied with SOM, pH, N content, etc. were selected to comprehensively explore the effects of OTC and Cd application on nitrification in different soil types. Although antibiotics and heavy metals have been frequently detected in many environments and its combined exposure significantly threaten the N cycle, the detailed ecological toxic effects of OTC and Cd on soil nitrification were currently unclear in soils. The purpose of this study was to reveal the effects of single or combined contamination of antibiotics and heavy metals on nitrification activity in different soil types. We hypothesized the following: (1) both single and combined OTC and Cd contamination could decrease net nitrification rates (NNRs) and AOA, AOB, due to immediate toxicity of OTC and Cd for broad range of microbes; (2) the inhibitory effects of OTC and Cd coexist complexes on soil nitrification processes might be greater than the effect of their single contaminants; (3) the inhibitory effects of OTC and Cd on nitrification and ammonia oxidizers might vary with soil types because of SOM and pH.

## Materials and methods

### Soils collection

**Figure 1:**
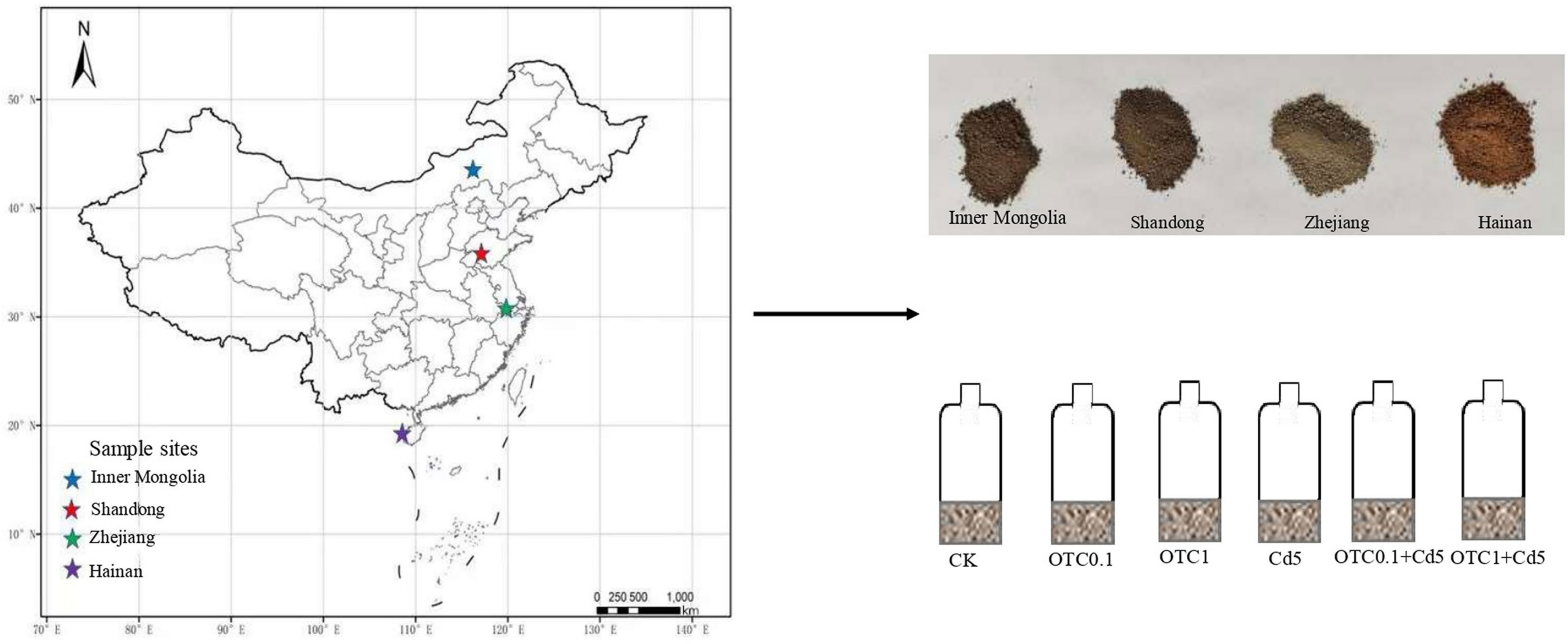
Sampling sites and soils in Inner Mongolia, Shandong, Zhejiang, and Hainan provinces from north to south of China. CK, control treatment without contamination; OTC0.1, OTC addition at 0.1 mg·kg^−1^; OTC1, OTC addition at 1 mg·kg^−1^; Cd5, Cd addition at 5 mg·kg^−1^; OTC0.1 + Cd5, OTC addition at 0.1 mg·kg^−1^ and Cd addition at 5 mg·kg^−1^; OTC1 + Cd5, OTC addition at 1 mg·kg^−1^, and Cd addition at 5 mg·kg^−1^.

The surface soils (0–20 cm) were sampled and were sieved (<2 mm) from Inner Mongolia (Chestnut soils), Shandong (Brown soils), Zhejiang (Paddy soils), and Hainan (Latosols) of China in June 2021 (Figure 1), then stored at 4°C prior to the incubation experiment. The vegetation type was *Leymus chinensis* with some *Stipa grandis* and *Cleistogenes squarrosa* in Inner Mongolia (Chestnut soils). The cropping systems in Shandong (Brown soils), Zhejiang (rice soils), and Hainan (Latosols) were winter wheat-summer corn rotation, rice-wheat rotation and rice-melon rotation, respectively. All the tested soils were uncontaminated soils, with the Cd and OTC contents below the limit of detection. Soils were sampled in an “S” pattern, with each plot being sampled at five random locations with a 5 cm diameter auger, and then mixed and combined to form a composite sample. The composite samples were transported to the laboratory at low temperature and passed through a 2 mm sieve for soil property analysis and incubation. The soil water content was measured after 24 h at 105°C. Soil pH was measured with a pH detector at a 2.5:1 (w:v) ratio of soil to distilled water. Soil organic carbon (SOC) content was analyzed by wet digestion with H_2_SO_4_-K_2_Cr_2_O_7_. The soil NH_4_^+^-N and NO_3_^−^-N were determined by continuous flow analyzer after extraction with 1 M KCl. The soil basic physical and chemical properties were listed in Table 1.

**Table 1:** Physicochemical properties of sampled soils.

Sample sites	Soil types	pH	Total nitrogen	Available phosphorous	Available potassium	Organic matter
			(g·kg^−1^)	(mg·kg^−1^)	(mg·kg^−1^)	(g·kg^−1^)
Inner Mongolia	Chestnut soils	7.17 ± 0.01a	1.59 ± 0.06b	25.19 ± 3.23a	182.33 ± 1.45a	18.46 ± 1.31b
Shandong	Brown soils	6.95 ± 0.02a	1.03 ± 0.04c	18.15 ± 3.48b	104.53 ± 2.17b	16.12 ± 1.81b
Zhejiang	Paddy soils	5.59 ± 0.01b	1.65 ± 0.02a	15.7 ± 2.79c	117.50 ± 1.25b	22.3 ± 2.45a
Hainan	Latosols	5.70 ± 0.01b	1.23 ± 0.04c	14.50 ± 2.42c	92.67 ± 2.71b	11.77 ± 2.94c

Values represent the means ± standard error for triplicate replicates. Different letters indicate significant differences with *p* < 0.05 between the groups.

The net nitrification rates (NNRs) were calculated using the following equation (Khanom et al., 2021):


(1) $$N=\frac{{\left(NO_{3}^{-}-N\right)}_{t_{2}}-{\left(NO_{3}^{-}-N\right)}_{t_{1}}}{t_{2}-t_{1}}\;$$

Where *N* represents the NNRs (mg·kg^−1^·d^−1^); (NO_3_^−^-N)_t2_ and (NO_3_^−^-N)_t1_ are the concentrations of NO_3_^−^-N at times 2 and 1, respectively.

The OTC with purity >95% and the CdCl_2_ 2.5 H_2_O with purity >99.95% were purchased from Aladdin Reagent Company. The OTC and Cd stock solution were prepared as aqueous solutions in sterile deionized water. Other chemicals used for the incubation were of analytical grade and purchased from Sinopharm Chemical Reagent Co., Ltd.

### Incubation experiment

Fresh soil (equivalent to 50.0 g dry weight) was pre-incubated at 40% water holding capacity in a 250 ml serum bottle for 7 days at 25°C in darkness before the incubation experiment. The levels of OTC and Cd addition were referenced to the residual concentrations in soils and animal manure according to the previous research (Li et al., 2010; Afzal et al., 2019; Guo et al., 2021). Specifically, the addition of OTC at 0.1 mg·kg^−1^ could be conducted to explore the toxic effects in lightly contaminated soils. OTC addition at 1 mg·kg^−1^ could be the threshold content of OTC contamination in combined contaminated soils and could be used to analyze the dose-effect relationship (Ma et al., 2016; Tang et al., 2020). Furthermore, the toxic effects of Cd at 5 mg·kg^−1^ on nitrification were proved to be more typical and reliable (Kavvadias et al., 2012; Afzal et al., 2019). Microcosms were established using seven treatments (each in triplicate) including (Ι) Control treatment without contamination (CK); (II) OTC addition at 0.1 mg·kg^−1^ (OTC0.1); (III) OTC addition at 1 mg·kg^−1^ (OTC1); (IV) Cd addition at 5 mg·kg^−1^ (Cd5); (V) OTC addition at 0.1 mg·kg^−1^ and Cd addition at 5 mg·kg^−1^ (OTC0.1 + Cd5); (VI) OTC addition at 1 mg·kg^−1^ and Cd addition at 5 mg·kg^−1^ (OTC1 + Cd5). OTC and Cd were dissolved in sterile water and added to each serum bottle at the concentrations above. The soil moisture was monitored by weight loss and adjusted to 60% of the water holding capacity. The consistent moisture content was maintained by periodically adding deionized water to compensate for any water loss during the entire incubation period. Soils were incubated at 25 ± 2°C in darkness and were collected at 0, 14, 28, and 56 days. Approximately 3 g of soil for molecular analysis was mixed and immediately stored at −80°C and the remaining soil was used to determine other properties.

### Soil DNA extraction, quantitative PCR, and high-throughput sequencing analysis

DNA was extracted from each soil sample (0.5 g) after incubation for 0 and 56 days with the Fast DNA kit (MP Biomedicals, United States) based on the manufacturer’s instruction. The extracted soil DNA samples were stored at −80°C for further analysis. The abundance of the functional genes was determined using an ABI Q5 real-time PCR system (Applied Biosystems, California, United States). The 20 μl reaction system included 10 μl of SYBR qPCR Master Mix (Vazyme), 0.4 μl primers, 1 μl of DNA template, and 8.6 μl of double-distilled water. The PCR primers were detailed in Table 2. The reaction conditions of AOA and AOB were as follows: pre-denaturation at 95°C for 30 s, denaturation at 95°C for 10 s, then 30 s at annealing temperatures (55°C for AOA-*amoA* gene or 57°C for AOB-*amoA* gene), extension at 72°C for 30 s, and the cycles were all 40. The amplification efficiency of functional gene copies was 88%–110% and R^2^ values were between 0.990 and 0.999.

**Table 2:** Primers and conditions used in this study.

Primer name	Primer sequence (5′-3′)	Target gene	References
Arch-*amoA*F	STA ATG GTC TGG CTT AGA CG	archaeal *amoA* gene	Francis et al. (2005)
Arch-*amoA*R	GCG GCC ATC CAT CTG TAT GT		
*amoA*-2R	CCC CTC KGS AAA GCC TTC TTC	bacterial *amoA* gene	Rotthauwe et al. (1997)

The AOA-*amoA* and AOB-*amoA* genes were sequenced on the Illumina Miseq PE300 (Illumina Inc., San Diego, CA, United States) at Shanghai Majorbio Bio-Pharm Technology Co., Ltd. platform using the same primers used for qPCR. For AOA, only one sequence direction was used for the downstream analysis, since amplified AOA *amoA* gene sequences with the primer set ArchamoAF/ArchamoAR had a length of 651 bp and thus did not have overlap (Chen et al., 2019; Lin et al., 2020). Raw reads were quality controlled using Trimmomatic software and spliced using FLASH. The sequences were OTU clustered based on 97% similarity using UPARSE (version 7.1 http://drive5.com/uparse/). In addition, chimeric sequences were checked and rejected using UCHIME. Raw sequence data were deposited into the NCBI Sequence Read Archive (SRA) database under accession number PRJNA874156 (AOB) and PRJNA874158 (AOA).

### Statistical analysis

Average, standard error, significance test and two-factor Analysis of Variance (ANOVA) were analyzed by SPSS 21.0. Significance was analyzed by the Duncan method with *p* < 0.05. Spearman’s rank correlations between NNRs and AOA, AOB *amoA* gene abundances were calculated in SPSS 21.0. The graphic drawing was performed on Origin 2021. A structural equation model (SEM) was constructed using IBM SPSS Amos 24.0 (AMOS IBM, United States) to evaluate the effects of contaminants on nitrification. Principal coordinates analysis (PCoA) based on Bray–Curtis distance matrices was carried out with the “vegan” package of R language (R version 3.3.1; R Core Team, 2013). The types of interactions between OTC and Cd combined contamination were analyzed by using the inhibition ration (IR).


(2) $$\mathrm{IR}=\frac{\mathrm{CK}-\mathrm{Treated population}}{\mathrm{CK}}\;$$

CK represents the *amoA* gene copies of the soil without OTC and/or Cd.

Treated population represents the *amoA* gene copies of the soil with OTC and/or Cd.

## Results

### Individual and combined effects of OTC and Cd on soil mineral N

To understand the effect of exogenous pollutants on nitrification process, soil mineral N under all treatments was monitored throughout the 56-day incubation period (Supplementary Figures S1, S2). The NH_4_^+^-N contents gradually and significantly decreased with incubation time regardless of OTC and Cd addition or not (Supplementary Figure S1). The individual and combined addition of OTC and Cd significantly increased NH_4_^+^-N concentrations at day 14 in all soils (Supplementary Figure S1). In addition, the NH_4_^+^-N contents showed no significant changes at days 28 and 56 compared with the control treatment in chestnut soils (Supplementary Figures S1A,D). The individual and combined addition of OTC and Cd exerted no significant effect on NH_4_^+^-N concentrations at day 28, while remarkably decreased NH_4_^+^-N concentrations at day 56 in brown soils (Supplementary Figure S1B). With respect to paddy soils, NH_4_^+^-N concentrations showed no significant change by individual and combined addition of OTC and Cd at days 28 and 56. OTC1 + Cd5 addition broadly increased NH_4_^+^-N content at day 28, OTC1 drastically increased NH_4_^+^-N content at day 56 (Supplementary Figure S1C). Similarly, NH_4_^+^-N concentrations had no significant change by individual and combined addition of OTC and Cd at days 28 and 56 in latosols. The combined addition of OTC and Cd significantly decreased NH_4_^+^-N concentrations at day 28 (Supplementary Figure S1D). The NO_3_^−^-N concentrations increased continuously and significantly during the 56-day incubation period (Supplementary Figure S2). Application of OTC and Cd significantly decreased the NO_3_^−^-N concentrations. The combined application of OTC and Cd showed a greater inhibitory effect on NO_3_^−^-N concentrations than OTC or Cd alone (Supplementary Figure S2). The inhibitory effects were significant at days 14 and 28 in northern alkaline soils (chestnut and brown soils) and were only remarkable at day 14 in southern acidic soils (paddy and latosols soils; Supplementary Figures S2A–D).

**Figure 2:**
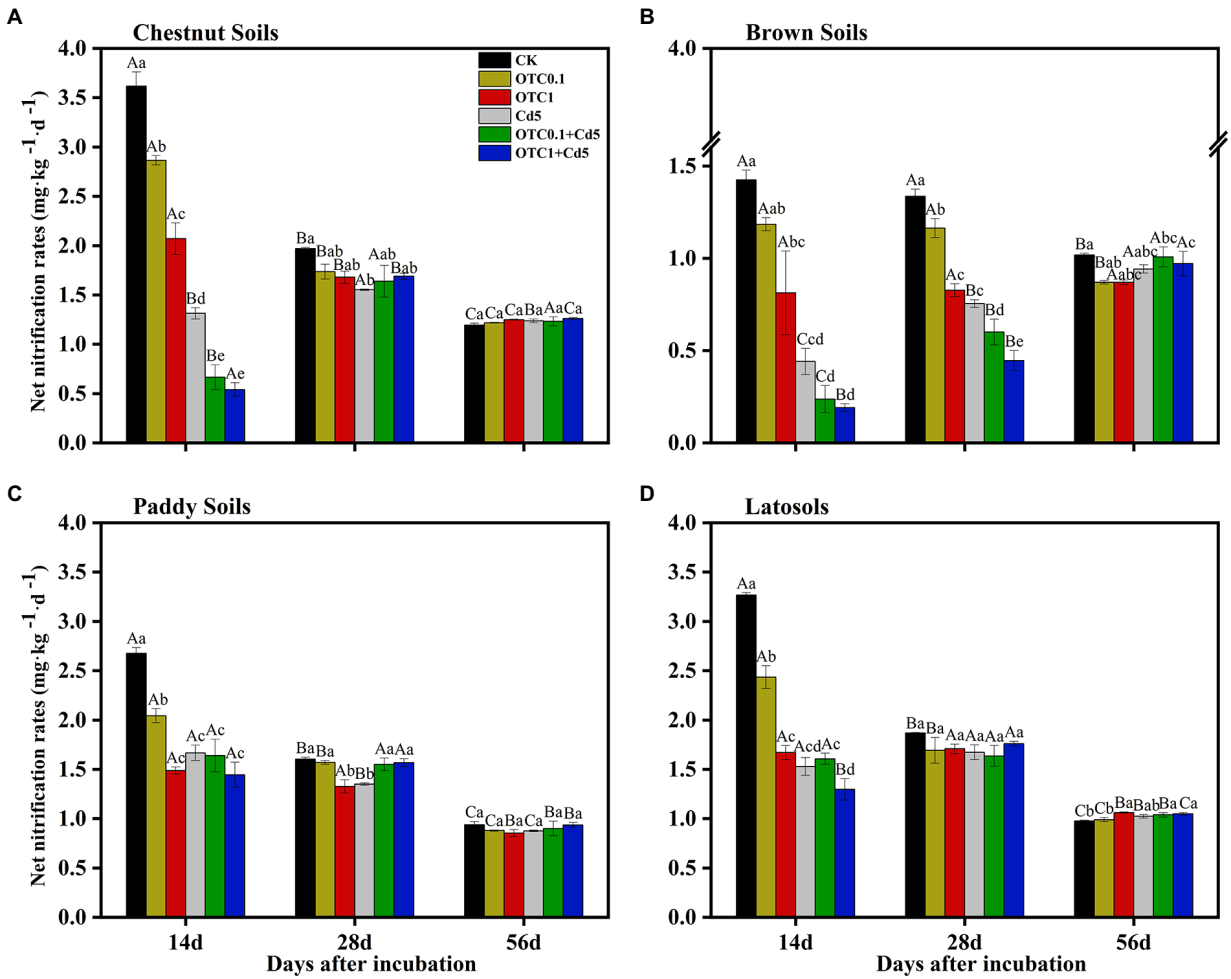
Dynamics of NNRs in chestnut soils **(A)**, brown soils **(B)**, paddy soils **(C)**, and latosols **(D)** under CK, OTC0.1, OTC1, Cd5, OTC0.1 + Cd5, and OTC1 + Cd5 treatments over the 56-day incubation. The vertical bars indicated the standard errors of the mean of triplicate samples. Different lowercase letters above the error bars indicated significant differences among treatments (*p* < 0.05). Different uppercase letters above the error bars indicated significant differences among sampling time points under the same treatment (*p* < 0.05). CK, control treatment without contamination; OTC0.1, OTC addition at 0.1 mg·kg^−1^; OTC1, OTC addition at 1 mg·kg^−1^; Cd5, Cd addition at 5 mg·kg^−1^; OTC0.1 + Cd5, OTC addition at 0.1 mg·kg^−1^ and Cd addition at 5 mg·kg^−1^; OTC1 + Cd5, OTC addition at 1 mg·kg^−1^ and Cd addition at 5 mg·kg^−1^.

NNRs were calculated to further reveal the effects of individual and combined pollution of OTC and Cd on nitrification activity (Figure 2). The pollution of OTC and Cd clearly decreased the NNRs compared with CK treatment in all soils (except brown soils; Figures 2A–D). The inhibitory effects were distinct at days 14 and 28 in northern alkaline soils (chestnut and brown soils) and were only remarkable at day 14 in southern acidic soils (paddy and latosols soils) compared with the control treatment. Additionally, the inhibitory effects were increased with increasing OTC concentration. Combined pollution of both OTC and Cd further stimulated the inhibitory effects.

### Individual and combined effects of OTC and Cd contamination on the *amoA* gene abundance

**Figure 3:**
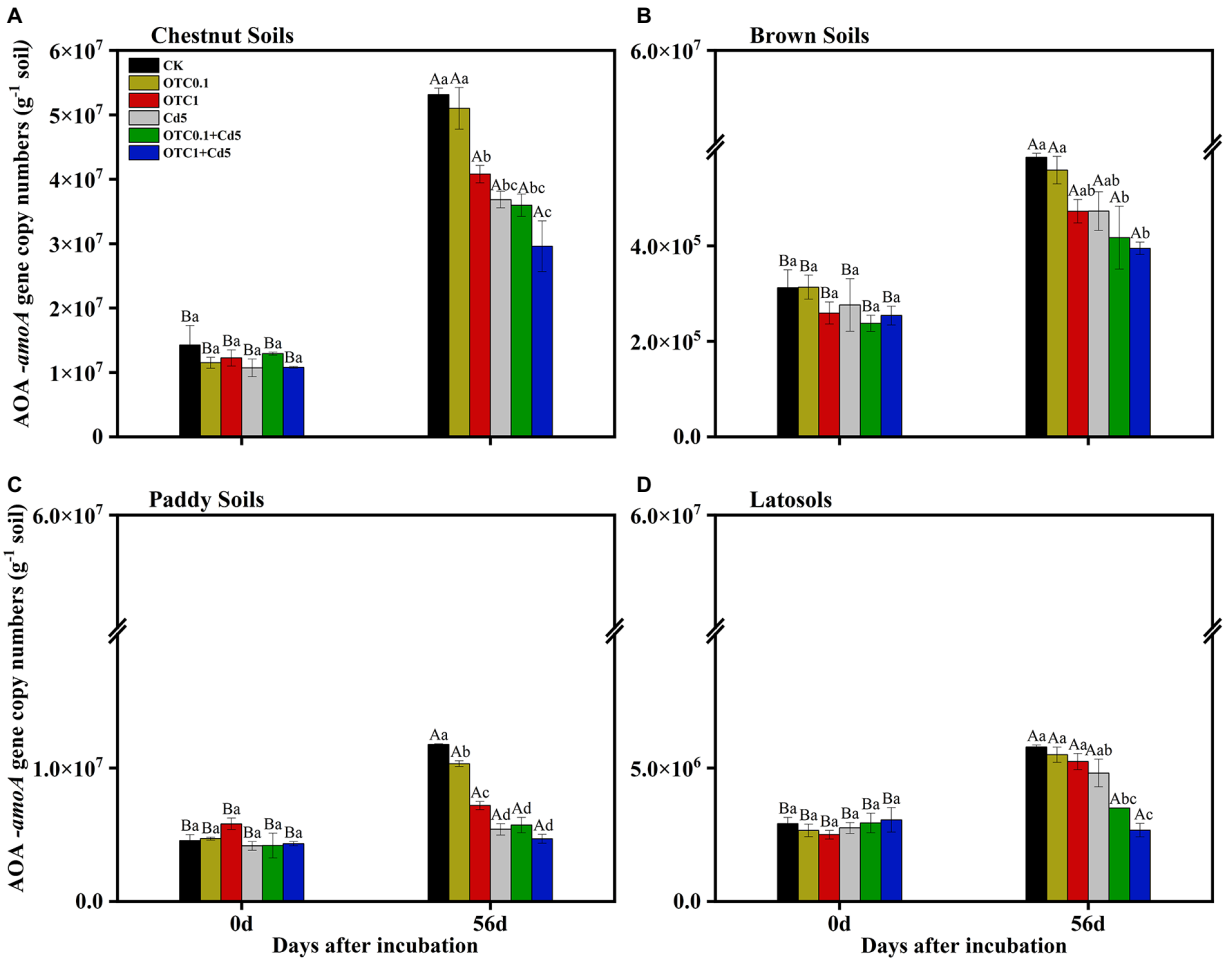
Abundance of AOA *amoA* gene in chestnut soils **(A)**, brown soils **(B)**, paddy soils **(C)**, and latosols **(D)** under CK, OTC0.1, OTC1, Cd5, OTC0.1 + Cd5, and OTC1 + Cd5 treatments at day 0 and day 56. The vertical bars indicated the standard errors of the mean of triplicate samples. Different lowercase letters above the error bars indicated significant differences among treatments (*p* < 0.05). Different uppercase letters above the error bars indicated significant differences among sampling time points under the same treatment (*p* < 0.05). CK, control treatment without contamination; OTC0.1, OTC addition at 0.1 mg·kg^−1^; OTC1, OTC addition at 1 mg·kg^−1^; Cd5, Cd addition at 5 mg·kg^−1^; OTC0.1 + Cd5, OTC addition at 0.1 mg·kg^−1^ and Cd addition at 5 mg·kg^−1^; OTC1 + Cd5, OTC addition at 1 mg·kg^−1^, and Cd addition at 5 mg·kg^−1^.

Generally, the copy numbers of AOA and AOB *amoA* genes increased over the whole incubation time in OTC and Cd-treated soils. The abundances of AOA *amoA* ranged from 2.96 × 10^7^ to 5.31 × 10^7^, 3.95 × 10^5^ to 5.86 × 10^5^, 4.70× 10^6^ to 1.18 × 10^7^, and 2.67 × 10^6^ to 5.78 × 10^6^ copies g^−1^ *d.w.s.* (dry weight soil) in the chestnut, brown, paddy, and latosols soils, respectively, from day 0 to day 56 (Figure 3). The AOA *amoA* gene copy numbers were significantly higher in chestnut soils than in other soils. Overall, the dynamics of AOA abundance resembled that of NNRs. Individual and combined pollution of OTC and Cd significantly decreased AOA *amoA* gene copy numbers. And the inhibitory effects were increased with the increasing of OTC concentration, especially in combined polluted soils.

**Figure 4:**
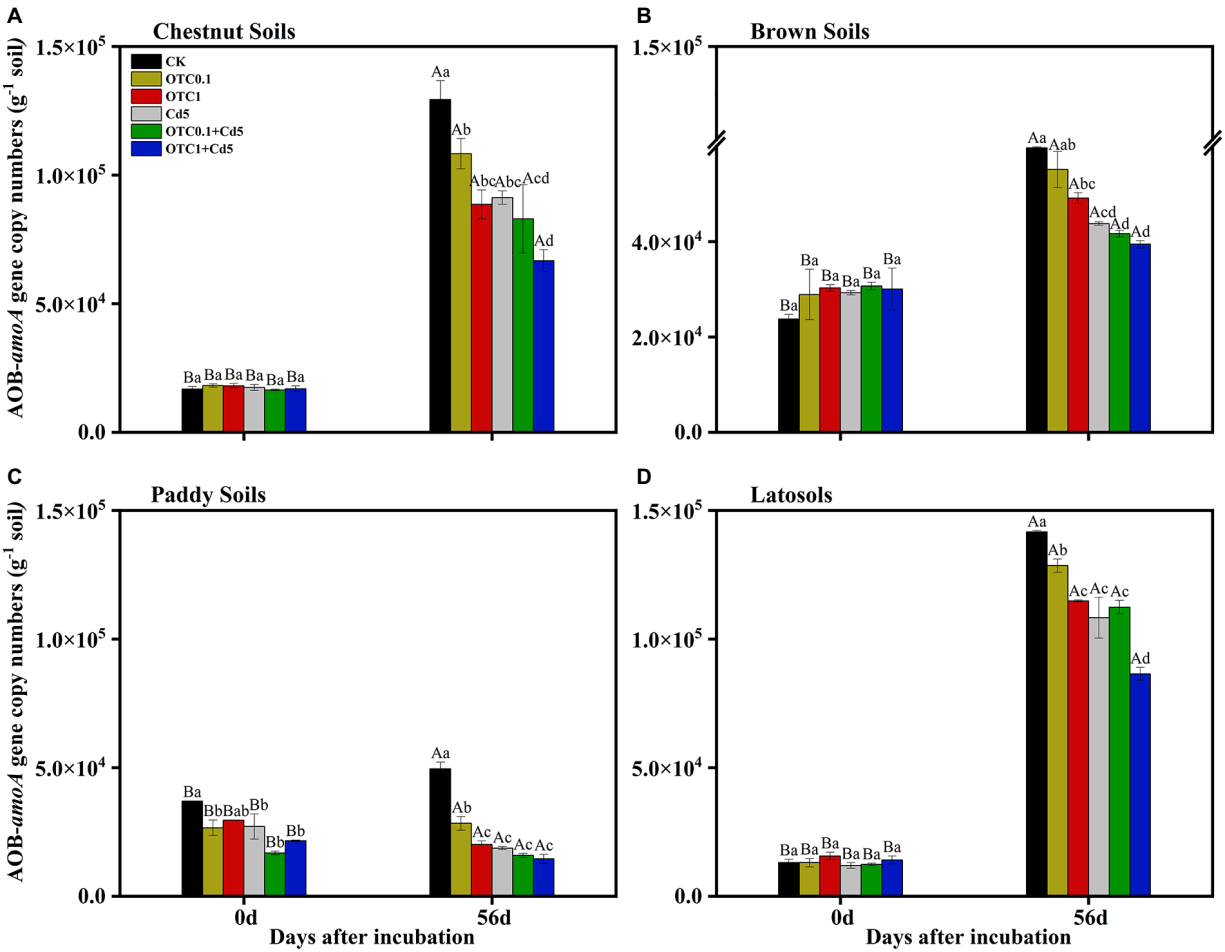
Abundance of AOB *amoA* gene in chestnut soils **(A)**, brown soils **(B)**, paddy soils **(C)**, and latosols **(D)** under CK, OTC0.1, OTC1, Cd5, OTC0.1 + Cd5, and OTC1 + Cd5 treatments at day 0 and day 56. The vertical bars indicated the standard errors of the mean of triplicate samples. Different lowercase letters above the error bars indicated significant differences among treatments (*p* < 0.05). Different uppercase letters above the error bars indicated significant differences among sampling time points under the same treatment (*p* < 0.05). CK, control treatment without contamination; OTC0.1, OTC addition at 0.1 mg·kg^−1^; OTC1, OTC addition at 1 mg·kg^−1^; Cd5, Cd addition at 5 mg·kg^−1^; OTC0.1 + Cd5, OTC addition at 0.1 mg·kg^−1^ and Cd addition at 5 mg·kg^−1^; OTC1 + Cd5, OTC addition at 1 mg·kg^−1^, and Cd addition at 5 mg·kg^−1^.

The copy numbers of the AOB *amoA* gene in the chestnut, brown, paddy and latosols soils ranged from 1.65 × 10^4^ to 1.29 × 10^5^, 2.37 × 10^4^ to 5.97 × 10^4^, 1.69 × 10^4^ to 4.95 × 10^4^, 1.24 × 10^4^ to 1.42 × 10^5^ copies g^−1^ *d.w.s.* from day 0 to day 56, respectively (Figure 4). The dynamics of AOB abundance followed the same trend with AOA *amoA* gene copy numbers. The inhibitory effects of pollution on AOB abundance followed the trend OTC1 + Cd5 > OTC0.1 + Cd5 > Cd5 > OTC1 > OTC0.1.

### Individual and combined effects of OTC and Cd contamination on AOA and AOB community structures

**Figure 5:**
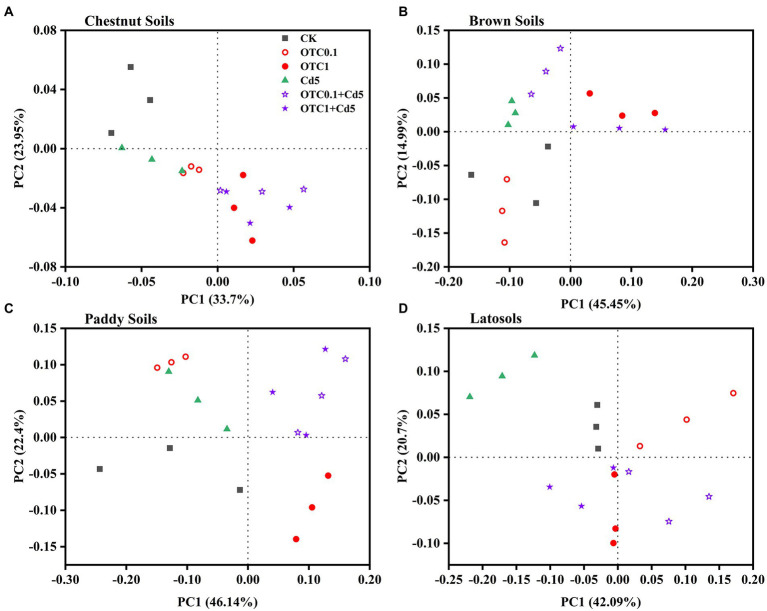
Principal coordinate analysis (PCoA) of the Bray–Curtis distance matrix representing differences in the community structure of AOA under CK, OTC0.1, OTC1, Cd5, OTC0.1 + Cd5, and OTC1 + Cd5 treatments in chestnut soils **(A)**, brown soils **(B)**, paddy soils **(C)**, and latosols **(D)**. CK, control treatment without contamination; OTC0.1, OTC addition at 0.1 mg·kg^−1^; OTC1, OTC addition at 1 mg·kg^−1^; Cd5, Cd addition at 5 mg·kg^−1^; OTC0.1 + Cd5, OTC addition at 0.1 mg·kg^−1^ and Cd addition at 5 mg·kg^−1^; OTC1 + Cd5, OTC addition at 1 mg·kg^−1^, and Cd addition at 5 mg· kg^−1^.

**Figure 6:**
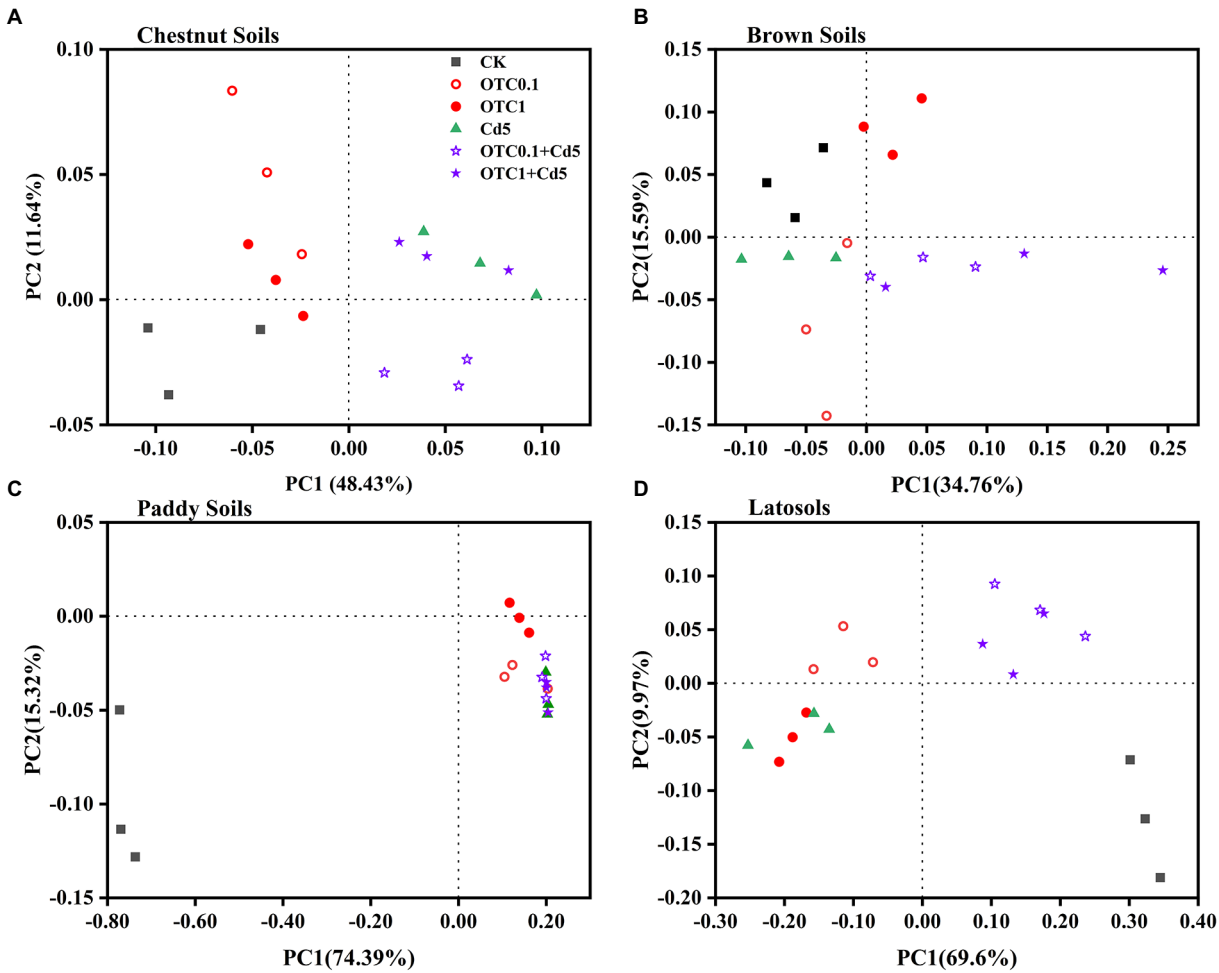
Principal coordinate analysis (PCoA) of the Bray–Curtis distance matrix representing differences in the community structure of AOB under CK, OTC0.1, OTC1, Cd5, OTC0.1 + Cd5, and OTC1 + Cd5 treatments in chestnut soils **(A)**, brown soils **(B)**, paddy soils **(C)**, and latosols **(D)**. CK, control treatment without contamination; OTC0.1, OTC addition at 0.1 mg·kg^−1^; OTC1, OTC addition at 1 mg·kg^−1^; Cd5, Cd addition at 5 mg·kg^−1^; OTC0.1 + Cd5, OTC addition at 0.1 mg·kg^−1^ and Cd addition at 5 mg·kg^−1^; OTC1 + Cd5, OTC addition at 1 mg·kg^−1^, and Cd addition at 5 mg· kg^−1^.

The principal coordinate analysis (PCoA) based on Bray-Curtis distance matrices was used to analyze the differences in the community structure of AOA and AOB among different treatments. The results showed that the AOA and AOB community patterns were significantly influenced by individual and combined pollution of OTC and Cd (Figures 5, 6). As for the AOA community, the first two principle components explained 57.65%, 60.44%, 68.54%, and 62.79% of the total variation of the AOA community composition in chestnut, brown, paddy soils, and latosols, respectively (Figure 5). The PCoA plots demonstrated distinct AOA community separations among treatments including OTC1, OTC0.1 + Cd5, OTC1 + Cd5 and other treatments (CK, Cd5, OTC0.1) along the first principle coordinates in chestnut soils and paddy soils (Figures 5A,C). Distinct separations were observed between high OTC treatments (OTC1, OTC1 + Cd5) and other treatments (CK, Cd5, OTC0.1, OTC0.1 + Cd5) along the first principle coordinates in brown soils (Figure 5B). Distinct separations were observed between low OTC treatments (OTC0.1, OTC0.1 + Cd5) and other treatments (CK, Cd5, OTC1, OTC1 + Cd5) along the first principle coordinates in latosols soils (Figure 5D).

With regards to the AOB community, the first two principle components explained 60.07, 50.35, 89.71, and 79.57% of the total variation of AOB community patterns in chestnut, brown, paddy, and latosols soils, respectively (Figure 6). The PCoA plots demonstrated distinct AOB community separations between Cd treatments (with or without OTC) and other treatments (CK, OTC0.1, OTC1) along the first principle coordinates in chestnut soils (Figure 6A). Distinct AOB community separations between treatments including OTC1, OTC0.1 + Cd5, OTC1 + Cd5 and other treatments (CK, Cd5, OTC0.1) along the first principle coordinates in brown soils (Figure 6B). No significant separations were observed in AOB communities in paddy soils among various treatments (Figure 6C). In latosols, individual OTC or Cd pollution significantly separated AOB community from combined pollution treatments (OTC0.1 + Cd5, OTC1 + Cd5) along the first principle coordinates (Figure 6D).

### Individual and combined effects of OTC and Cd contamination on nitrification

The effects of soil type and pollution on NNRs, AOA and AOB abundance were analyzed by two-factor ANOVA analysis (Table 3). Either Soil type or contamination treatments exerted significant effects on AOA (*p* < 0.01, R^2^ = 0.990) and AOB (*p* < 0.01, R^2^ = 0.990). In addition, significant effects of soil types alone (*p* < 0.01, R^2^ = 0.911), and pollution alone on NNRs (*p* < 0.05, R^2^ = 0.911) were also observed. The interactive effects of soil types and pollution were significant on soil nitrification and associated functional microbes. Moreover, OTC and Cd exerted antagonistic inhibitory effects on AOB in all tested soils. As for AOA, the interaction of OTC and Cd was synergistic in Latosols soils and antagonistic in chestnut and paddy soils (Table 4).

**Table 3:** Two-factor ANOVA based on soil types and pollution treatments.

	NNRs at 56 days			AOA *amoA* at 56 days			AOB *amoA* at 56 days		
Factor	df	F	*p*	df	F	*p*	df	F	*p*
Soil types	3	150.868	<0.01^**^	3	1475.664	<0.01^**^	3	1396.397	<0.01^**^
Contamination treatments	5	2.59	<0.05^*^	5	30.385	<0.01^**^	5	124.521	<0.01^**^
Soil types and contamination treatments	15	1.795	<0.05^*^	15	11.829	<0.01^**^	15	7.697	<0.01^**^

* represents significance at *p* < 0.05.

** extremely indicates significance at *p* < 0.01.

df, degrees of freedom.

**Table 4:** Interaction types of OTC and Cd on AOA and AOB at day 56.

Microbe	Treatments	Inhibition rate (%)				Type of interaction			
		Chestnut soils	Brown soils	Paddy soils	Latosols	Chestnut soils	Brown soils	Paddy soils	Latosols
AOA	OTC0.1	4.04	4.61	12.28	5.01				
	OTC1	23.25	19.31	38.89	9.44				
	Cd5	30.7	19.26	54.06	16.88				
	OTC0.1 + Cd5	32.34	28.75	51.3	39.51	A	S	A	S
	OTC1 + Cd5	44.35	32.57	60.05	53.88	A	A	A	S
AOB	OTC0.1	16.29	7.66	42.76	9.28				
	OTC1	31.51	17.67	59.28	18.95				
	Cd5	29.48	26.65	62.15	23.57				
	OTC0.1 + Cd5	35.85	30.16	67.95	20.66	A	A	A	A
	OTC1 + Cd5	48.44	33.9	70.63	38.97	A	A	A	A

If the combined inhibition rate of OTC and Cd is greater than the sum of the individual inhibition rates of each contaminant, the type of interaction is considered as synergistic effect (S), or is considered as antagonistic effect (A).

**Figure 7:**
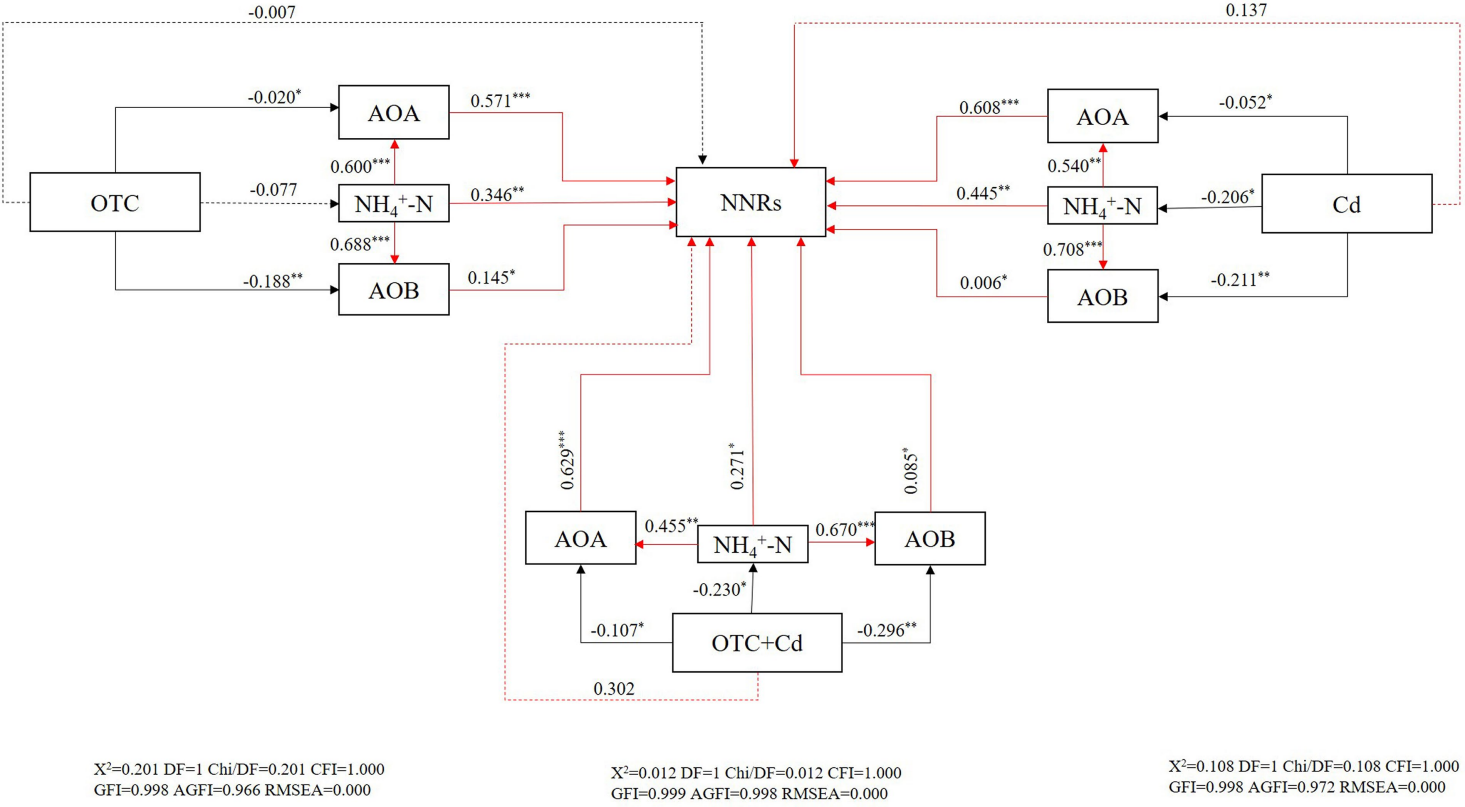
Structural equation models (SEM) showing the direct and indirect effects of contamination treatments on AOA, AOB, NH_4_^+^-N, and NNRs. ^*^ indicates significant differences at *p* < 0.05, ^**^ indicates significant differences at *p* < 0.01, and ^***^ indicates significant differences at *p* < 0.001. Numbers on arrows indicate significant standardized path coefficients. Positive effects are represented by red lines, while negative effects are represented by black lines. Continuous and dashed lines represent significant and nonsignificant relationships, respectively.

Correlation analysis revealed that NNRs showed strong and positive relationships with AOA (r = 0.546, *p <* 0.01; r = 0.460, *p <* 0.05; r = 0.382, *p <* 0.01) and AOB (r = 0.619, *p <* 0.01; r = 0.576, *p <* 0.01; r = 0.492, *p <* 0.01) under OTC pollution, Cd pollution, as well as under combined OTC and Cd contamination, respectively. SEM analysis was constructed to further investigate the direct or indirect effects of pollutants on NNRs (Figure 7). The results indicated the directly and negatively effects of combined and single OTC and Cd treatments on AOB and AOA *amoA* genes. The inhibitory effects of pollutants on AOB were stronger than those on AOA. Furthermore, both AOA and AOB showed a significantly positive influence on NNRs. Individual pollution of OTC inhibited NNRs by influencing ammonia oxidizers and Cd contaminant (presence or absence of OTC) had indirect effects on nitrification activity *via* inhibiting mineral N and ammonia oxidizers.

## Discussion

### OTC inhibited soil nitrification process by inhibiting ammonia oxidizers

The addition of OTC (1 mg·kg^−1^) alone significantly decreased NO_3_^−^-N levels compared to the control treatment in brown and paddy soils during the whole incubation period, while this effect was only observed at days 14 and 28 in chestnut and latosols soils (Supplementary Figure S2). Likewise, previous researches also revealed that 1 mg·kg^−1^ OTC inhibited nitrification and decreased NO_3_^−^-N concentrations in long-term non-farmed soils (Liu et al., 2020) and silt loam soils (Cao et al., 2016). The addition of 3,300 mg·kg^−1^ OTC significantly inhibited nitrification after incubation for 94 days in pristine sandy loam soils (Piotrowska-Seget et al., 2008). Trifonova et al. (2021) found that OTC (200 mg·kg^−1^) inhibited urease activity but had no significant effect on nitrification activity in sod-podzolic soils during 90 days incubation. These discrepancies could be attributed to the fact that the active N nutrient substrate could be depleted in soils, and the antibiotics degraded as the half-life values of 1 and 100 mg·kg^−1^ OTC were 31.5 and 33 days, respectively (Liu et al., 2020; Tang et al., 2020).

OTC1 significantly inhibited AOB and AOA in all tested soils, while OTC0.1 inhibited AOA and AOB only in paddy soils (Figures 3, 4). In addition, AOB was inhibited more strongly than AOA under OTC1 in brown and latosols soils, and under OTC0.1 in chestnut and latosols soils. Combined with the results that AOA showed remarkably higher abundance than AOB, we speculated that AOA was more tolerant than AOB to OTC pollution. Similarly, previous results reported that OTC addition as well as the individual and combined contaminations of SM2 and Cu inhibited AOB more serious than AOA, and thus AOB was more sensitive to contaminants than AOA (Wang et al., 2019; Tang et al., 2020). The possible reason might be due to the fact that archaea shared peptidoglycan-free cell walls and ether lipids membranes, which were more resistant to antibiotics (Conkle and White, 2012; Tang et al., 2020). Besides, the inhibitory effects of OTC on ammonia oxidizers could be explained by the fact that the nitrobacteria were typically Gram-negative bacteria, whose growth was inhibited by OTC (Chopra and Roberts, 2001). In contrast, some studies found that OTC addition could stimulate ammonia oxidizers rather than inhibit their growth. For example, Cao et al. (2016) revealed that 1 mg·kg^−1^ OTC significantly increased AOB abundance and inhibited AOA *amoA* gene in silt loam soils. However, Wang L. et al. (2018) proposed that AOA was facilitated by low concentrations of OTC (23 mg·kg^−1^) and inhibited by high concentrations (92 and 368 mg·kg^−1^). This could result from the antibiotic content in the soil exceeded the concentration threshold even cause complete inhibition of microbial populations. In addition, AOA and AOB share different cell structure, hence their tolerance and sensitivity to same environment are different. Besides, our results revealed that AOA (r = 0.546, *p* < 0.01) and AOB (r = 0.619, *p* < 0.01) *amoA* genes were strongly and positively correlated with NNRs under OTC treatments. Combined with the SEM analysis results that OTC pollution indirectly inhibited nitrification activity by inhibiting AOA and AOB abundance (Figure 7), we concluded that both AOA and AOB dominated nitrification in OTC-contaminated soils. In addition, the results revealed that the inhibition effects of OTC on nitrification were increased with the increasing concentrations of OTC, which was further supported by previous researchers who determined dose-dependence of the inhibitory effects on AOA and AOB (Wang J. et al., 2018; Wang L. et al., 2018).

### Cd inhibited soil nitrification process by influencing mineral N and ammonia oxidizers

In this study, Cd addition alone significantly inhibited the NNRs in chestnut, brown and paddy soils at 14 and 28 days (Figure 2). Moreover, Cd alone decreased the AOB *amoA* gene copy numbers in four soils and decreased AOA abundance only in chestnut and paddy soils (Figures 3, 4). Both correlation analysis and SEM analysis indicated that Cd inhibited nitrification activity through influencing mineral N and AOA/AOB (Figure 7). During the later stages of incubation, nitrification gradually recovered, which could be explained by the screening effect of Cd stress on Cd-resistant species, making the newly selected microorganisms more resistant to the toxic effects of Cd than the native species, resulting in the gradual recovery of soil microbial functions and enzyme activities. For example, Ruyters et al. (2010) found the gradual development of a Zn-tolerant nitrifying community, whose ability to adapt to Zn stress in the soil increased after a 12-month Zn-polluted incubation. The results that Cd contamination decreased the N cycle microbial abundance and slowed the N transformation (Oka and Uchida, 2018; Afzal et al., 2019) might arise from the fact that Cd^2+^ could replace the active site in ammonia monooxygenase (AMO), thus preventing AMO from oxidizing NH_3_ to NH_2_OH (Radniecki and Ely, 2008; Kapoor et al., 2015). It was also reported that Cd stress (8 mg·kg^−1^) produced sustained stimulatory effect on both AOB and AOA, with AOA being most strongly stimulated in the later incubation stage (Zhao et al., 2021). Some studies demonstrated that low levels of Cd (2 and 5 mg·kg^−1^) addition would stimulate nitrification, while inhibition effects were only found at high levels of Cd addition (10 and 20 mg·kg^−1^) in alluvial soils (Chen et al., 2001). Furthermore, Salam et al. (2020) reported that Cd contamination significantly affected the structure and function of soil microbial communities, resulting in massive loss of non-adapted native microorganisms, as well as changed the soil physicochemical properties. Thus, the response of nitrification process to Cd addition varied with Cd concentrations and soil types (Liu et al., 2019).

### Combined pollution of OTC and Cd inhibited soil nitrification process by influencing mineral N and ammonia oxidizers

Combined contamination of OTC and Cd obviously inhibited AOA and AOB *amoA* gene copies as well as NNRs in all soils. And the response time and extent of nitrification activity to the combined contamination of OTC and Cd varied with soil types. For example, combined addition of OTC (0.1 and 1 mg·kg^−1^) and Cd decreased the NNRs in brown soils at day 56, while the NNRs content wasn’t significantly changed in chestnut and paddy soils (Figure 2). This could be attributed to the fact that interaction effects of antibiotics and heavy metals were affected by soil properties, and the complexation of antibiotics and heavy metals varied with soils, thus altering the sorption and desorption of antibiotics and heavy metals (Xu et al., 2015; Zhang et al., 2018). It had been reported that coexistence of TC and Cd (II) reduced each other’s mobility in alkaline environment such as calcareous and saline soils, while Cd (II) had no effect on TC mobility in acidic soils (Du et al., 2021). In addition, the absorption of antibiotics by SOM can be significantly prolonged and enhanced (Kong et al., 2012; Álvarez-Esmorís et al., 2020). And SOM was the dominant adsorbent for Cd and decreased Cd availability or mobilization (Filipović et al., 2018; Chen et al., 2020). In addition, OTC0.1 significantly inhibited AOA in paddy soils while 0.1 mg·kg^−1^ of OTC had no significant toxic effects on AOA in other tested soils. Correlation analysis and SEM analysis further confirmed that combined pollution of OTC and Cd inhibited soil nitrification process by influencing mineral N and ammonia oxidizers (Figure 7). This was explained by antibiotics and heavy metals inhibited the replication and transcription of the function gene *amoA* of AOB and AOA, thus inhibiting the oxidation of NH_4_^+^-N and reducing soil NO_3_^−^-N concentrations (Kapoor et al., 2015; Tong et al., 2015; Tang et al., 2020). Hence, we speculated that the variation of pH and organic matter content in different soils could be the main reason for the different toxic effect. And an antagonism between OTC and Cd on AOB was observed in all tested soils. However, OTC and Cd had synergistic inhibitions on AOA in brown and latosols soils (Table 4). Wang J. et al. (2018) found enrofloxacin (ENR) and Cd interaction effects mainly antagonistic on AOB, which might be related to various factors such as concentrations, incubation time of mixture contaminants as well as soil properties. In addition, the combined pollution of OTC and Cd had greater negative effects on nitrification than those with the single contaminant. This resulted from the relative inhibition of dissipation and increased sorption of OTC by Cd, and OTC-Cd complexes might be formed by OTC and Cd, and the complexes showed greater toxic effects than the containments used alone. Jia et al. (2008) found that the TC-Cu^2+^ complexes formed in the presence of TC had greater affinity for the soil surface than Cu^2+^ alone, which enhanced the adsorption of Cu^2+^ in red soils and paddy soils. Thus, the antibiotics bound metals *via* multiple coordination sited to produce the complex (Zhang et al., 2012; Tong et al., 2015). This result was further supported by Tang et al. (2020), who revealed that combined pollution of antibiotics and heavy metals had greater toxicity than single pollution in soil nitrification process. Similarly, Kong et al. (2006) found that OTC and Cu also had significant negative effects on soil microbial community function, with the negative effects were more significant in the co-existence of both contaminants.

Additionally, our results also suggested that AOA *amoA* gene abundance was higher than AOB *amoA* gene abundance and the inhibitory effects of OTC and Cd on AOB were greater than on AOA in all tested soils. This was consistent with previous studies showing that the AOA *amoA* gene copies were higher than AOB *amoA* gene copies in many soils, and AOB was more sensitive than AOA to the antibiotics and heavy metals combined treatments (Leininger et al., 2006; Wang L. et al., 2018; Wang et al., 2019; Tang et al., 2020). Moreover, the combined toxicity of two pollutants was significantly greater than the toxicity of either pollutant alone and was most pronounced in the early stage during incubation, but the inhibitory effects decreased with incubation time. The results were partially consistent with previous studies (Wang J. et al., 2018; Wang L. et al., 2018), which might be due to the degradation of OTC and the reduction of Cd potency, and the concentration of antibiotics and bioavailable heavy metals at day 56 might be below the limit for inhibiting the activity of ammonia-oxidizing microorganisms. The inhibition effect of OTC and Cd on AOB in latosols soils enhanced with increasing OTC levels, as well as on NNRs in brown soils, indicated that the dose-dependent relationship also existed in the combined OTC and Cd pollution (Ma et al., 2016; Wang L. et al., 2018). In addition, due to the large differences in the properties of different antibiotics and heavy metals, only typical types of antibiotics and heavy metals were selected for this study. Therefore, the results of this study could provide a reference for the mechanism of the action of single or combined contamination of heavy metals and antibiotics on soil N cycle. Moreover, an experiment investigated the structure and form of complex contaminants after OTC and Cd exposure to soils would be considered in the near future to comprehensively understand the morphology and structure of contaminants in different soil types after OTC and Cd pollution.

## Conclusion

The effects of individual and combined OTC and Cd on soil nitrification were examined in the study. The single and combined application of OTC and Cd showed different levels of significant inhibitory effect on soil nitrification. The combined toxicity of OTC and Cd was generally greater than that from either contaminant acting alone. Moreover, the toxic effects of single and combined contaminants on nitrification were significantly enhanced with increasing concentrations of OTC, and varied with soil types because of soil pH and SOM. The interaction of OTC and Cd on AOB and AOA was mainly antagonistic. Both AOA and AOB dominated in nitrification in the individual and combined contaminated soils, and AOB was more sensitive than AOA to both OTC and Cd toxicity. OTC inhibited nitrification activity by inhibiting ammonia oxidizers, and Cd (with or without OTC) inhibited nitrification through influencing mineral N and ammonia oxidizers.

## Data availability statement

The authors confirm that the data supporting the findings of this study are available within the article and its Supplementary materials.

## Author contributions

HP and YZ provided the idea of this study. XC, HP, and YZ designed the study. XC completed most of the experimental procedures, data analysis and writing the manuscript. JH performed the correlation analysis and SEM analysis in the revised manuscript. WZ, HZ, JL, and JH analyzed the soil physicochemical properties. HP and YZ supervised the work. HP, YZ, YL, HW, and QY revised the manuscript. All authors contributed to the article and approved the submitted version.

## Funding

This research was funded by the Major Science and Technology Innovation Projects in Shandong Province (2021CXGC010804).

## Conflict of interest

The authors declare that the research was conducted in the absence of any commercial or financial relationships that could be construed as a potential conflict of interest.

## Publisher’s note

All claims expressed in this article are solely those of the authors and do not necessarily represent those of their affiliated organizations, or those of the publisher, the editors and the reviewers. Any product that may be evaluated in this article, or claim that may be made by its manufacturer, is not guaranteed or endorsed by the publisher.

## Supplementary material

The Supplementary material for this article can be found online at: https://www.frontiersin.org/articles/10.3389/fmicb.2022.1062703/full#supplementary-material
