# Supplementary material for: Individual and combined contamination of oxytetracycline and cadmium inhibited nitrification by inhibiting ammonia oxidizers
Source: Front Microbiol. 2022 Dec 1;13:1062703. doi: 10.3389/fmicb.2022.1062703 (PMC9751337; doi:10.3389/fmicb.2022.1062703)
Supplement: Supplementary file 1 [file Data_Sheet_1.docx]

***Supplementary Material***

**Individual and combined contamination of oxytetracycline and cadmium inhibited nitrification by inhibiting** **ammonia oxidizers**

**Xiaoxu Cao, Wei Zhao, He Zhang, Jitong Lin, Jingying Hu, Yanhong Lou, Hui Wang, Quangang Yang, Hong Pan ^*^, Yuping Zhuge ^*^**

National Engineering Research Center for Efficient Utilization of Soil and Fertilizer Resources, College of Resources and Environment, Shandong Agricultural University, Daizong Road, Tai’an 271018, China

**^*^ Correspondence:** Hong Pan, Yuping Zhuge

Tel: +86- 538- 8243918

FAX: +86- 538- 8242250

E-mail addresses: panhong6239@163.com; zhugeyp@sdau.edu.cn

Add: College of Resources and Environment, Shandong Agricultural University, Tai’an City, Shandong, China

**Figure captions**

**Figure S1.** Dynamics of NH_4_^+^–N in chestnut soils (a), brown soils (b), paddy soils (c), and latosols (d) under CK, OTC0.1, OTC1, Cd5, OTC0.1+Cd5, and OTC1+Cd5 treatments over the 56-day incubation. The vertical bars indicated the standard errors of the mean of triplicate samples. Different lowercase letters above the error bars indicated significant differences among treatments (*P* < 0.05). Different uppercase letters above the error bars indicated significant differences among sampling time points under the same treatment (*P* < 0.05). CK, control treatment without contamination; OTC0.1, OTC addition at 0.1 mg· kg^-1^; OTC1, OTC addition at 1 mg· kg^-1^; Cd5, Cd addition at 5 mg· kg^-1^; OTC0.1+Cd5, OTC addition at 0.1 mg· kg^-1^ and Cd addition at 5 mg· kg^-1^; OTC1+Cd5, OTC addition at 1 mg· kg^-1^ and Cd addition at 5 mg· kg^-1^.

**Figure S2.** Dynamics of NO_3_^-^–N in chestnut soils (a), brown soils (b), paddy soils (c), and latosols (d) under CK, OTC0.1, OTC1, Cd5, OTC0.1+Cd5, and OTC1+Cd5 treatments over the 56-day incubation. The vertical bars indicated the standard errors of the mean of triplicate samples. Different lowercase letters above the error bars indicated significant differences among treatments (*P* < 0.05). Different uppercase letters above the error bars indicated significant differences among sampling time points under the same treatment (*P* < 0.05). CK, control treatment without contamination; OTC0.1, OTC addition at 0.1 mg· kg^-1^; OTC1, OTC addition at 1 mg· kg^-1^; Cd5, Cd addition at 5 mg· kg^-1^; OTC0.1+Cd5, OTC addition at 0.1 mg· kg^-1^ and Cd addition at 5 mg· kg^-1^; OTC1+Cd5, OTC addition at 1 mg· kg^-1^ and Cd addition at 5 mg· kg^-1^.


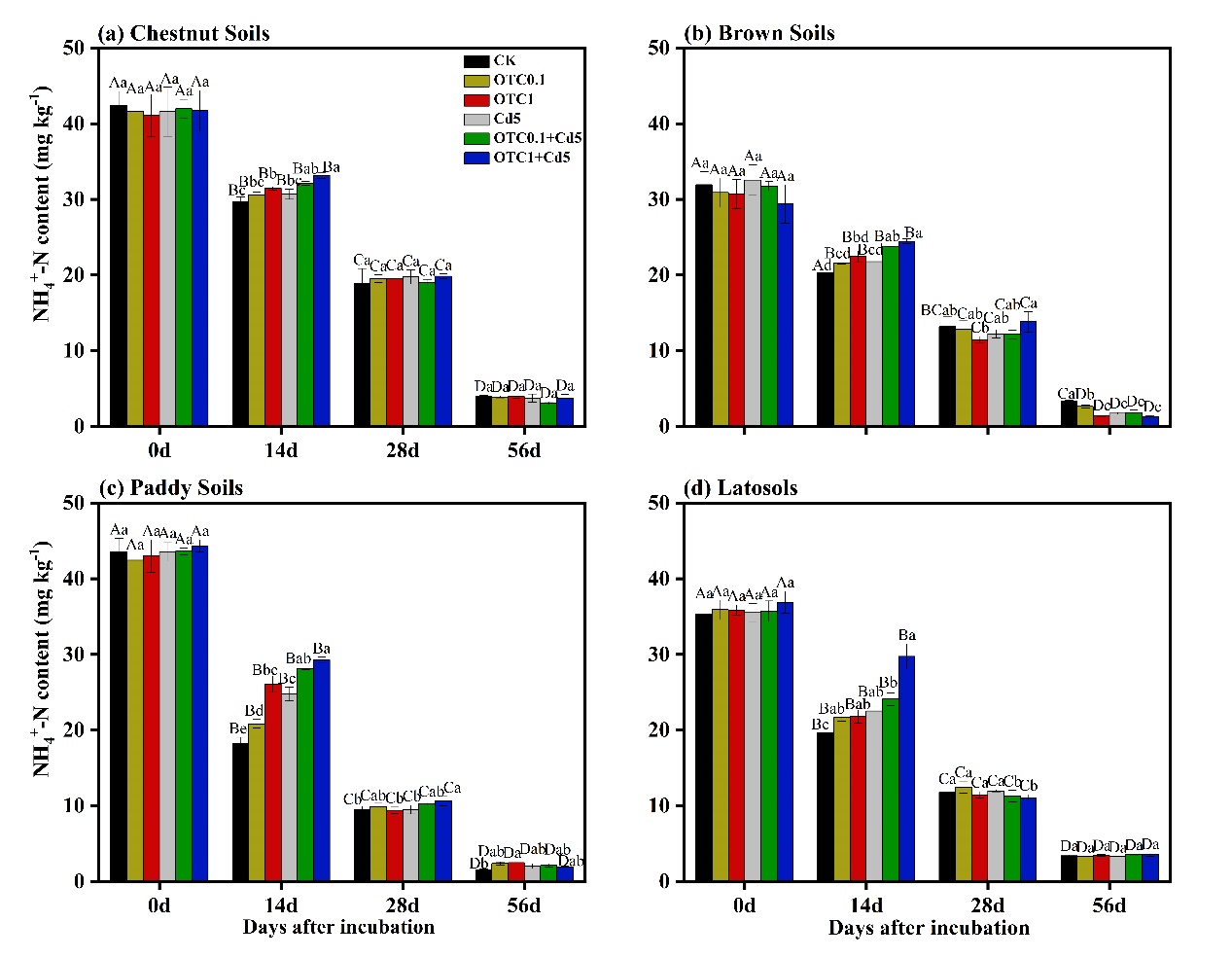


**Figure S1.**

**
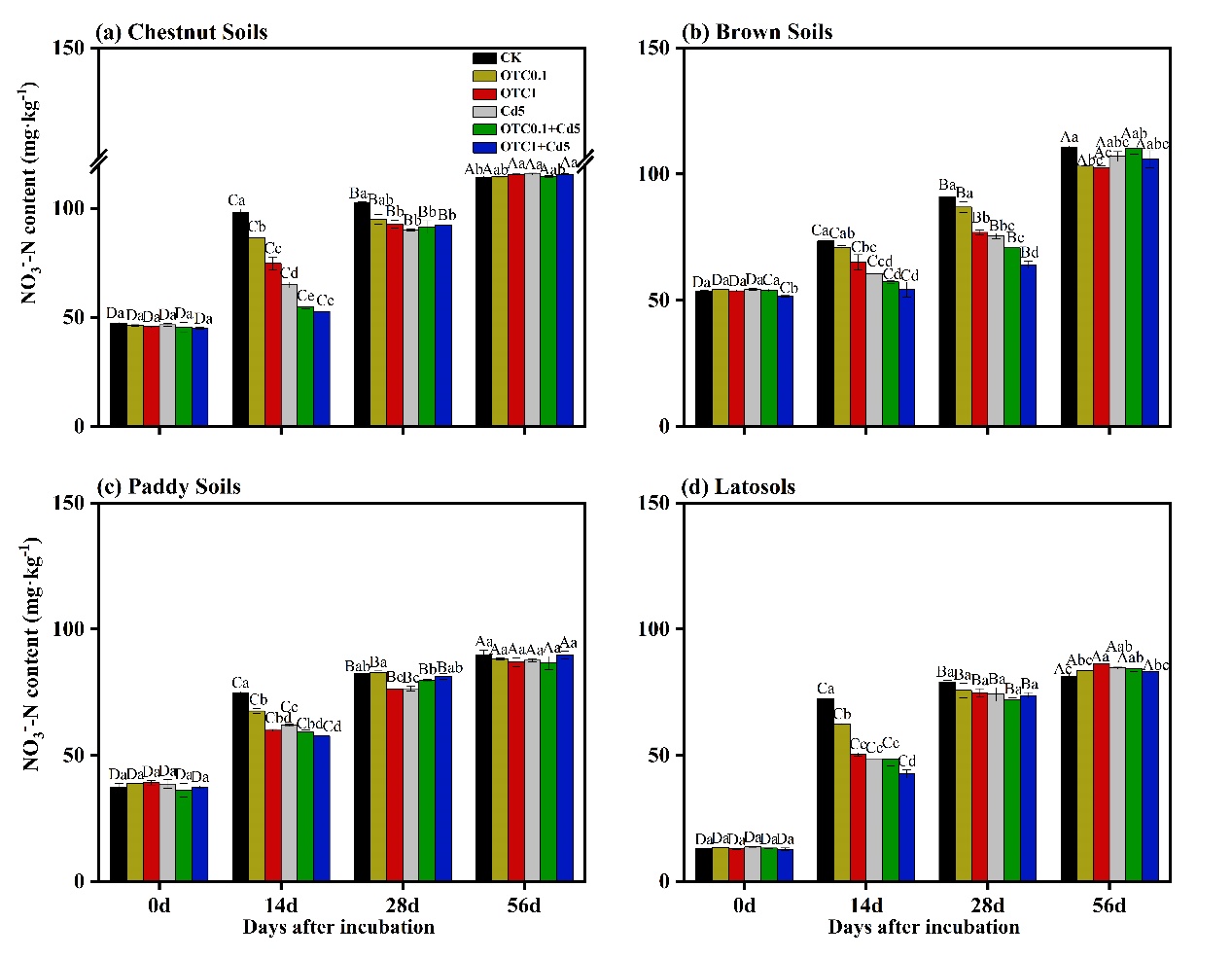
**

**Figure S2.**
